# BIRC6 Protein, an Inhibitor of Apoptosis: Role in Survival of Human Prostate Cancer Cells

**DOI:** 10.1371/journal.pone.0055837

**Published:** 2013-02-08

**Authors:** Christopher G. Low, Iris S. U. Luk, Dong Lin, Ladan Fazli, Kuo Yang, Yong Xu, Martin Gleave, Peter W. Gout, Yuzhuo Wang

**Affiliations:** 1 The Vancouver Prostate Centre, Vancouver General Hospital and Department of Urologic Sciences, the University of British Columbia, Vancouver, British Columbia, Canada; 2 Department of Experimental Therapeutics, BC Cancer Agency, Vancouver, British Columbia, Canada; 3 Department of Urology, The Second Hospital of Tianjin Medical University, Tianjin Institute of Urology, Tianjin, China; The Chinese University of Hong Kong, Hong Kong

## Abstract

**Background:**

BIRC6 is a member of the Inhibitors of Apoptosis Protein (IAP) family which is thought to protect a variety of cancer cells from apoptosis. The main objective of the present study was to investigate whether BIRC6 plays a role in prostate cancer and could be useful as a novel therapeutic target.

**Methods:**

BIRC6 expression in cell lines was assessed using Western blot analysis and in clinical samples using immunohistochemistry of tissue microarrays. The biological significance of BIRC6 was determined by siRNA-induced reduction of *BIRC6* expression in LNCaP cells followed by functional assays.

**Results:**

Elevated BIRC6 protein expression was found in prostate cancer cell lines and clinical specimens as distinct from their benign counterparts. Increased BIRC6 expression was associated with Gleason 6–8 cancers and castration resistance. Reduction of BIRC6 expression in LNCaP cells led to a marked reduction in cell proliferation which was associated with an increase in apoptosis and a decrease in autophagosome formation. Doxorubicin-induced apoptosis was found to be coupled to a reduction in BIRC6 protein expression.

**Conclusion:**

The data suggest a role for BIRC6 in prostate cancer progression and treatment resistance, and indicate for the first time that the *BIRC6* gene and its product are potentially valuable targets for treatment of prostate cancers.

## Introduction

Prostate cancers usually present as androgen-dependent tumors, susceptible to growth arrest/apoptosis induced by androgen ablation therapy [1]. Although initially effective, androgen ablation frequently leads to the development of castration-resistant (androgen-independent) prostate cancer, which is generally also resistant to other available treatments. As such, castration resistance commonly marks the end stage form of prostate cancer and is the major obstacle in disease management [1]. Development of castration-resistant prostate cancer is characteristically associated with marked increases in resistance to apoptosis, a major death pathway for drug action [1]–[3]. Apoptosis resistance resulting from up-regulation of anti-apoptotic genes and their products is thought to be a key contributor in the development of castration resistance, as well as general resistance to anti-cancer treatments. Elucidating the role of anti-apoptotic genes/proteins in the progression of prostate cancer is therefore likely to lead to improvements in the treatment of refractory disease.

The Inhibitors of Apoptosis Protein (IAP) family has been reported to play a role in apoptosis resistance in a variety of cancer cell lines and is characterized by the presence in the proteins of one to three copies of a Baculoviral IAP Repeat (BIR) domain. The IAPs have been demonstrated to bind to and inhibit a variety of pro-apoptotic factors, thereby effectively suppressing apoptosis induced by a wide range of effectors, including chemotherapeutics and irradiation [4]. The BIR domain is essential for interaction of the IAPs with pro-apoptotic factors, including caspases. The caspases are a family of cysteine-aspartic acid-specific proteases, present in a pro-form which, once activated via cleavage, is responsible for degradation of death substrates such as poly-ADP-ribose polymerase (PARP) thereby triggering apoptosis. Cleaved caspase-3 and cleaved PARP can be readily detected by Western blot analysis and are commonly used as markers for apoptosis [5].

Apoptosis is often associated with autophagy, a process involving lysosomal degradation of a cell's own components [6]. It involves packaging of proteins and organelles within autophagosomes, followed by fusion with lysosomes leading to degradation of the proteins and organelles. The role of autophagy in the development of cancer and its treatment is complex, since there is evidence that autophagy can promote and suppress cancer growth [7]. Inhibition of autophagy by disruption of essential autophagy genes has been shown to promote tumorigenesis and hence autophagy can have a tumor-suppressive effect [8]–[11]. However, there is increasing evidence that autophagy can act as a survival mechanism for cancer cells in response to a wide range of stresses, including treatment with anti-cancer agents [7].

To detect autophagic activity in cultured cells, Western blot detection of LC3B-II is often used. LC3B-II is specifically associated with autophagosomes and levels of LC3B-II have been demonstrated to correlate with the number of autophagosomes within cells [12]–[15]. However, since LC3B-II is degraded upon autophagosome-lysosome fusion, LC3B-II levels offer only a snapshot of the number of autophagosomes in cells at one time-point and do not indicate an up-regulation or down-regulation of autophagy in its entirety [13], [15]. Accordingly, a decrease in the numbers of autophagosomes in a cell can occur by a decrease in autophagosome formation or an increase in autophagosome degradation. Detection of other critical autophagy proteins like Beclin-1 can offer further insight into the activation of autophagy within these cells. This protein is involved in both the signaling pathway activating autophagy and in the initial step of autophagosome formation [12], [16]. Currently there is no evidence suggesting a role for IAPs in the regulation of autophagy in humans.

The BIRC6 protein is at 528 kDa, an unusually large member of the IAP family. It consists of a single N-terminal BIR domain and a C-terminal ubiquitin-conjugating (UBC) domain; the latter has chimeric E2/E3 ubiquitin ligase activity as well as anti-apoptotic activity [17]. Through its BIR domain, BIRC6 is capable of binding to and inhibiting active caspases, including caspases-3, 6, 7 and 9 and such interactions have been shown to underlie BIRC6’s ability to inhibit the caspase cascade and ultimately apoptosis [17]. Through its UBC domain, BIRC6 facilitates proteasomal degradation of pro-apoptotic proteins caspase-9 [18], SMAC/DIABLO [18], [19] and HTRA2/OMI [17], [20]. BIRC6 is also a critical regulator of cytokinesis and hence plays an important role in cell proliferation [21].

Recent evidence supports a widespread role for BIRC6 in conferring apoptosis resistance to cancer cells, as indicated by *in vitro* studies with cells from gliomas [22], lung cancers [23], cervical cancers [19], [21], [24], [25], fibrosarcomas [18], [24], osteosarcomas [21], breast cancers [24], [26] and colon cancers [27]. In breast and lung cancer cells, apoptosis triggered by the loss of BIRC6 expression has been demonstrated to involve p53 stabilisation [23], [26]. BIRC6 expression in clinical cancer samples has been observed for colorectal cancer [28] and childhood *de novo* acute myeloid leukemia (AML) [29]. In the latter, elevated expression of *BIRC6* mRNA was associated with an unfavourable response to chemotherapy and poor relapse-free survival rates [29]. A role for BIRC6 in prostate cancer, however, has not been reported.

In the present study, analysis of human prostate cancer cell lines and clinical specimens showed marked elevations in BIRC6 expression by the malignant cells/tissues as distinct from their benign counterparts. In particular, increased BIRC6 expression was associated with Gleason 6–8 cancers and castration resistance. Furthermore, siRNA-induced knockdown of BIRC6 led to a marked reduction in cell proliferation of LNCaP prostate cancer cells. Taken together, the results suggest that BIRC6 represents a novel therapeutic target for treatment of refractory prostate cancer.

## Materials and Methods

### Materials

Chemicals, solvents, and solutions were obtained from Sigma-Aldrich Canada Ltd, Oakville, ON, Canada, unless otherwise indicated.

### Clinical Prostate Cancer Tissues

Specimens were obtained from patients, with their informed consent, following a protocol approved by the Clinical Research Ethics Board of the University of British Columbia and the BC Cancer Agency. Gleason-graded tissue microarrays (TMAs) were used consisting of 35 benign prostate specimens, 6 prostatic-intraepithelial neoplasia specimens and 157 radical prostatectomy prostate cancer specimens. The cancer specimens consisted of 74 Gleason score 6, 23 Gleason score 7, 43 Gleason score 8, 2 Gleason score 9, and 15 Gleason score 10 tissues that had not been subjected to neo-adjuvant hormone therapy and 10 prostate cancer tissues that had been subjected to neo-adjuvant hormone therapy and had progressed to castration-resistant disease. The latter 10 tissues had Gleason scores of 8 (n = 6) and 10 (n = 4) prior to therapy. Samples for TMA construction had been selected randomly from collections at the Vancouver Prostate Centre, Vancouver General Hospital (supplied by the Department of Pathology, University of British Columbia, Vancouver, BC, Canada). Tissue preparation and TMA construction were performed as described [30].

### Immunohistochemistry

Immunohistochemical analyses were performed as described [31] using a rabbit polyclonal anti-BIRC6 antibody (Novus Biologicals, Littleton, CO; NB110-40730) at a 1∶100 dilution. All sections used for immunohistochemistry were counterstained with 5% (w/v) Harris hematoxylin.

### BIRC6 Protein Scoring

BIRC6 staining of tissues was evaluated by a pathologist and given a score of 0, 1, 2 or 3, representing no BIRC6 staining, weak, moderate and strong BIRC6 staining intensity, respectively. Percent positivity (as a measure of staining frequency) was calculated for each group based on the number of sections with staining intensities of ‘1′ or higher.

### Cell Lines

Six human prostate cancer cell lines (LNCaP; PC3; PC3-M; DU145; C42; VCaP), two benign prostatic cell lines (BPH1, RWPE1) and two cell lines known to express BIRC6 (OVCAR-8, HeLa) were maintained in media supplemented with FBS (10%), penicillin G (100 IU/mL) and streptomycin (100 µg/mL) in a humidified incubator at 37°C and 5% CO_2_ [22]. Cancer cells were cultured using RPMI-1640 medium, BPH1 cells were cultured using DMEM, and RWPE1 cells using Keratinocyte-SFM (Gibco-BRL; Burlington ON, Canada). Cell lines were obtained from the American Type Culture Collection (ATCC, Manassas, VA). To determine the effect of apoptosis on BIRC6 expression in LNCaP cells, 600,000 cells were seeded in 6-well plates and incubated overnight and then incubated with doxorubicin (1 µg/mL).

### Western Blotting

Cell lysates were prepared using cell lysis buffer (1% NP-40, 0.5% sodium deoxycholic acid). For detection of BIRC6 (528 kDa), 10 µg whole cell lysate was run on a two-part (5% and 12.5%) SDS-polyacrylamide gel. Gels were cut, and BIRC6 was electrotransferred to a PVDF membrane in tris (25 mM), glycine (191.5 mM), methanol (10%), SDS (0.05%), using a semi-dry transfer apparatus. Membranes were probed for BIRC6 with rabbit polyclonal anti-BIRC6 antibody (1∶500; Novus Biologicals). For detection of PARP, caspase-3, LC3B-I, LC3B-II and Beclin-1 protein expression, 5–15 µg of whole cell lysate was run on 10 or 12.5% SDS-polyacrylamide gels and, following protein transfer, membranes were probed using rabbit anti-PARP and anti-Caspase-3 antibodies (1∶1000; Cell Signaling; Beverly, MA), rabbit anti-LC3B antibodies (0.85∶1000; Abcam, Cambridge, MA) and rabbit anti-Beclin-1 antibodies (1∶1000; Santa Cruz Biotechnology, Inc., Santa Cruz, CA). Actin or vinculin were used as loading controls and detected on membranes using rabbit anti-actin polyclonal antibody (1∶2000; Sigma-Aldrich) and mouse anti-vinculin antibody (1∶3000; Sigma-Aldrich).

### Small Interfering RNA (siRNA) and Cell Transfection

Custom siRNAs synthesized by Dharmacon (Lafayette, CO) and known to target *BIRC6* had the following sequences: siRNA-1, sense, 5′-GUU-UCA-AAG-CAG-GAU-GAU-G-dTdT-3′ [23] and siRNA-2, sense, 5′-CUC-AGG-AGA-GUA-CUG-CUC-A-dTdT-3′ [21]. Non-targeting siRNA (siGENOME Non-Targeting Smartpool; Dharmacon) was used as control. To examine the effect of the siRNAs on BIRC6 protein expression, LNCaP cells were plated in 6-well plates in antibiotic-free RPMI-1640 medium supplemented with fetal bovine serum (10%). After 24 h, the cells were transfected with 100 nM siRNA in lipofectamine 2000 reagent (Invitrogen; Burlington, ON) following the manufacturer’s instructions. Vehicle control and non-targeting siRNA were applied to replicate cell cultures.

### MTT Viability Assay

Twenty-five thousand cells were seeded per well of a 24-well dish and transfected with siRNA-2. At 0, 24, 48 and 72 h following transfection, 50 µL of MTT (5 mg/mL) was added to each well and cultures were incubated in a humidified incubator at 37°C and 5% CO_2_ for 4 h. Five hundred µL of 20% SDS solution was then added to each well and incubated overnight at room temperature in the absence of light. Samples (100 µL) were then transferred to 96-well plates. Absorbance was measured at 570 nm.

### Cell Cycle

The cell cycle distribution of LNCap cells was determined by flow cytometry of propidium iodide (PI) stained cells. Cells were cultured in RPMI-10% FBS medium and subsequently treated with *BIRC6* siRNA. Forty-eight hours after treatment with siRNA, cells were fixed by 70% ethanol and then stored at 4°C overnight. Fixed cells were washed once with PBS. After centrifugation, the cells were stained with 10 ug/ml propidium iodide (PI), 1 mg/ml RNase and 0.1% Triton X-100 for 30 mins on ice. DNA histograms were obtained by analyzing 10,000 cells. The proportion of cells in G1, S, and G2+ M of the cell cycle was determined.

### Annexin-V Assays

Apoptosis was measured by fluorescence-activated cell sorter (FACS) analysis with annexin-V conjugated with fluorescein isothiocyanate (annexin-V-FITC) (BD Biosciences PharMingen) for early apoptosis and propidium iodide (PI) for late apoptosis staining following the manufacturer's protocol. Cells were cultured in RPMI-10% FBS medium and subsequently treated with *BIRC6* siRNA. Forty-eight hours after treatment with siRNA, cells were harvested, washed with cold PBS and then resuspended in 1X Binding Buffer (BD PharMingen, San Diego, CA) at a concentration of ∼1×10^6^ cells/mL. Cell suspensions (100 µL; ∼1×10^5^ cells) were transferred to new tubes, and 5 µL Annexin V–FITC and 5 µL PI aliquots were added. The cells were incubated in the dark for 15 min at 21°C. Annexin–FITC fluorescence was measured in the FL1 channel (using a 530/30 band pass filter) and PI in FL3 channel (660/20 BP filter band pass filter). Ten thousand events were collected. Apoptosis was assessed by counting the percentage of AnnexinV-positive cells. Data are shown as means ± SD of triplicate culture.

### LC3-GFP Puncta Formation Assay and Quantification

LNCaP cells were seeded on coverslips in 6-well plates and transfected with 100 nM non-target siRNA or BIRC6 siRNA-2 the next day using Oligofectamine. Forty-eight hours after siRNA transfection, cells were transfected with 3 μg of LC3-GFP per well by XtremeGENE (Roche) and cells were fixed by 4% paraformaldehyde 27 hours after transfection. LC3-GFP puncta formation was induced 6 hours prior to fixation by treatment with 10 uM chloroquine in serum free medium. After fixation, coverslips were mounted with DAPI mounting solution. Fluorescent images were taken by confocal microscope. Quantification of autophagic cells: Autophagic cells were referred to as cells containing more than 15 LC3-GFP puncta. Percentage of autophagic cells was calculated as the number of autophagic cells divided by the number of LC3-GFP positive cells × 100.

### Statistical Analysis

The Student’s *t*-test was used and results with a *P*-value <0.05 were considered significant.

## Results

### Elevated BIRC6 Protein Levels in Human Prostate Cancer Cell Lines

**Figure 1:**
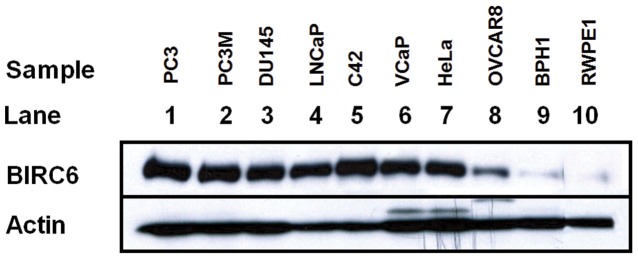
BIRC6 protein expression is elevated in prostate cancer cells in comparison with benign prostate cells. BIRC6 protein expression in malignant prostate cells (Lanes 1–6), positive control malignant cervical and ovarian cells (Lanes 7–8) and benign prostate cells (Lanes 9–10).

Western blotting revealed strong BIRC6 protein expression in all prostate cancer cell lines examined (Fig. 1). In contrast, only low levels of BIRC6 protein expression were detected in BPH1 and RWPE1 benign prostate cell lines. Moderate to strong BIRC6 expression was detected in OVCAR8 and Hela cells as reported by others [17], [22]. Results are representative of three independent experiments.

### Elevated BIRC6 Protein Levels in Gleason Scored Clinical Prostate Cancer Tissues

**Figure 2:**
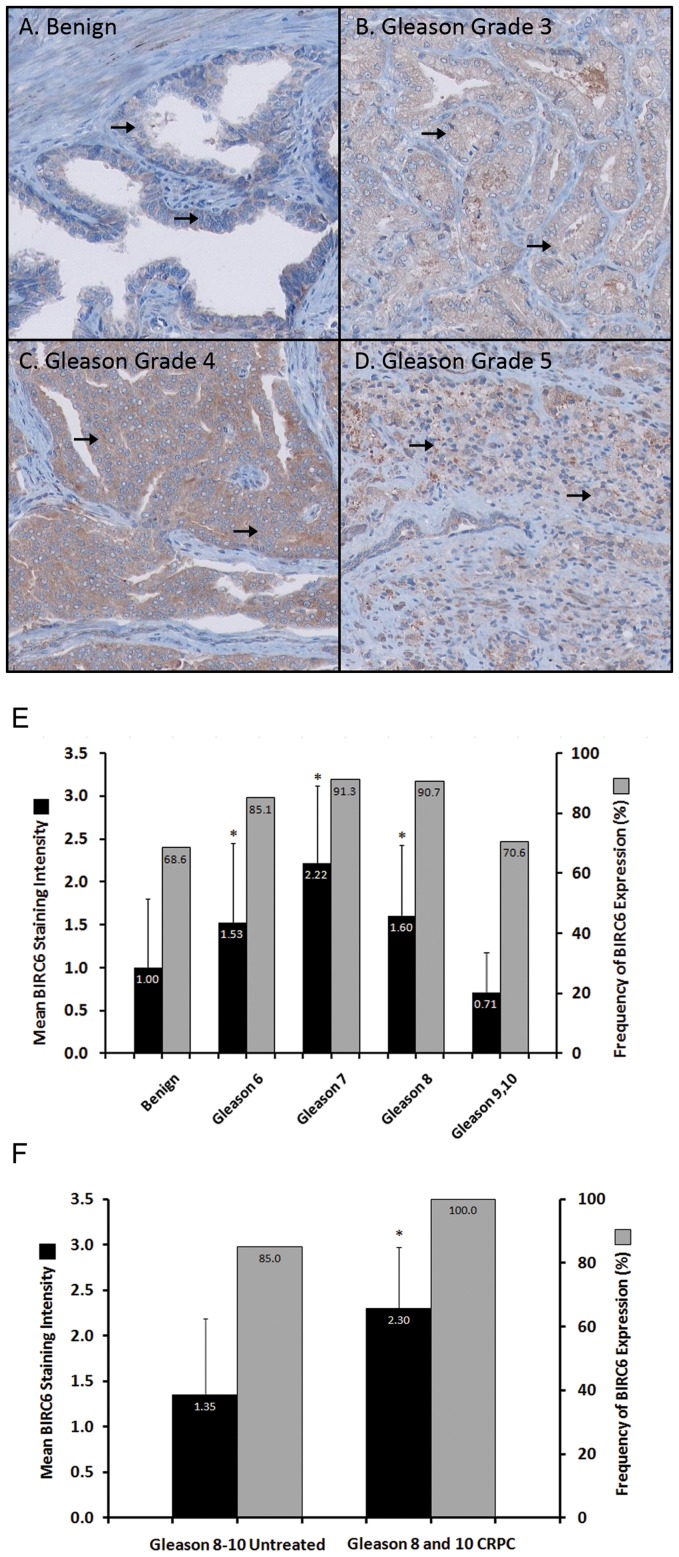
BIRC6 protein expression in TMA sections of prostate tissue samples. **A**, arrows indicating weak cytoplasmic BIRC6 staining in benign prostatic luminal cells (score ‘1′). **B**, arrows indicating moderate cytoplasmic BIRC6 staining in Gleason grade 3 prostate cancer cells (score ‘2′). **C**, arrows indicating strong cytoplasmic BIRC6 staining in Gleason grade 4 prostate cancer cells (score ‘3′). **D**, arrows indicating weak cytoplasmic BIRC6 staining in Gleason grade 5 prostate cancer cells (score ‘1′). **E**, Black bars representing mean BIRC6 staining intensity in benign prostate epithelium (n = 35) and Gleason score 6 (n = 74), 7 (n = 23), 8 (n = 43) and 9–10 (n = 17) patient prostate cancer tissues. Grey bars representing frequency of BIRC6 expression (score ≥1). Elevated staining intensities were observed in Gleason score 6 (*P = 3.24×10−3), 7 (*P = 4.45×10−6) and 8 (*P = 1.63×10−3) prostate cancer tissues as compared to benign prostate epithelium. Error bars depict standard deviations. **F**, Black bars representing mean BIRC6 staining intensity in untreated high grade (Gleason score 8–10) prostate cancer tissues (n = 60) and castration-resistant prostate cancer (CRPC) tissues which had developed from Gleason score 8–10 cancers following neo-adjuvant hormone therapy (n = 10). Grey bars representing frequency of BIRC6 expression (score ≥1). Castration-resistant prostate cancer tissues showed a significantly higher mean BIRC6 staining intensity than untreated high grade prostate cancer tissues (*P = 1.38×10−3). Error bars depict standard deviations.

Clinical prostate tissue sections, morphologically categorized into benign tissue and cancers of Gleason score 6–8 and 9–10, were stained and scored for BIRC6 expression. Positive cytoplasmic staining for BIRC6 was, generally, low in benign epithelium, substantially more intense in well differentiated Gleason grade 3 and strongest in poorly differentiated Gleason grade 4 prostate cancer tissues (Fig. 2A–D). Gleason grade 5 tissues generally expressed lower levels of BIRC6 staining relative to the other prostate cancer tissues, similar to benign tissues. Sections containing both benign tissue and cancer tissue (Gleason grade 3) showed strong positive BIRC6 staining in the malignant epithelium and absent or weak expression in the benign epithelium (data not shown). Mean staining intensities of PIN were slightly elevated compared to benign epithelium, although not significantly elevated (data not shown). In the Gleason scored tissues (Fig. 2E), expression of BIRC6 was low for benign tissues and rose steadily, peaking in Gleason score 7 cancers. Expression in Gleason score 8 cancers was lower and a further drop to levels similar to those of benign tissues was seen for Gleason score 9–10 specimens. The mean staining intensities for each group were 1.00±0.80 S.D. for benign epithelium and 1.53±0.92 S.D. (*P* = 3.24×10^−3^) for Gleason score 6, 2.22±0.90 S.D. (*P* = 4.45×10^−6^) for Gleason score 7 and 1.60±0.82 S.D. (*P* = 1.63×10^−3^) for Gleason score 8 prostate cancer tissues. The intensity of BIRC6 expression in Gleason score 9–10 prostate cancer tissues was similar to that of benign tissues (0.71±0.47 S.D.; *P* = 0.10). Also, the low intensity of expression observed in Gleason score 9–10 prostate cancer tissues was reflective of the large proportion of typically weak BIRC6-expressing Gleason grade 5 cancers found in this group.

Gleason score 6, 7 and 8 malignant epithelia more frequently expressed BIRC6 (staining intensity score ≥1) than benign prostate epithelium (Fig. 2E). All malignant tissues showed a high frequency of BIRC6 expression coupled to high BIRC6 intensity, except for Gleason score 9–10 tissues which showed a high frequency of BIRC6 expression coupled to a low average intensity. Taken together, the data suggest that prostate cancer progression from benign to Gleason score 8 prostate cancers is associated with elevations in BIRC6 protein expression.

### Elevated BIRC6 Protein Levels in Castration-resistant Clinical Prostate Cancer Tissues

Tissue sections from patients’ prostate cancers (Gleason score 8–10) which had progressed to castration resistance following neo-adjuvant hormone therapy (n = 10) were stained and scored for BIRC6 expression and compared with sections of high grade prostate cancers (Gleason score 8–10) which had not been subjected to neo-adjuvant hormone therapy (n = 60). The mean staining intensity for BIRC6 was significantly higher in castration-resistant prostate cancers than in untreated controls (2.30±0.67 S.D. and 1.35±0.84 S.D. respectively; *P* = 1.38×10^−3^) (Fig. 2F). BIRC6 expression frequency was very high in both types of tissue. The data suggest that development of castration-resistant prostate cancer is associated with elevations in BIRC6 protein expression.

### Reduction of BIRC6 Expression Decreases Prostate Cancer Cell Viability and Proliferation

**Figure 3:**
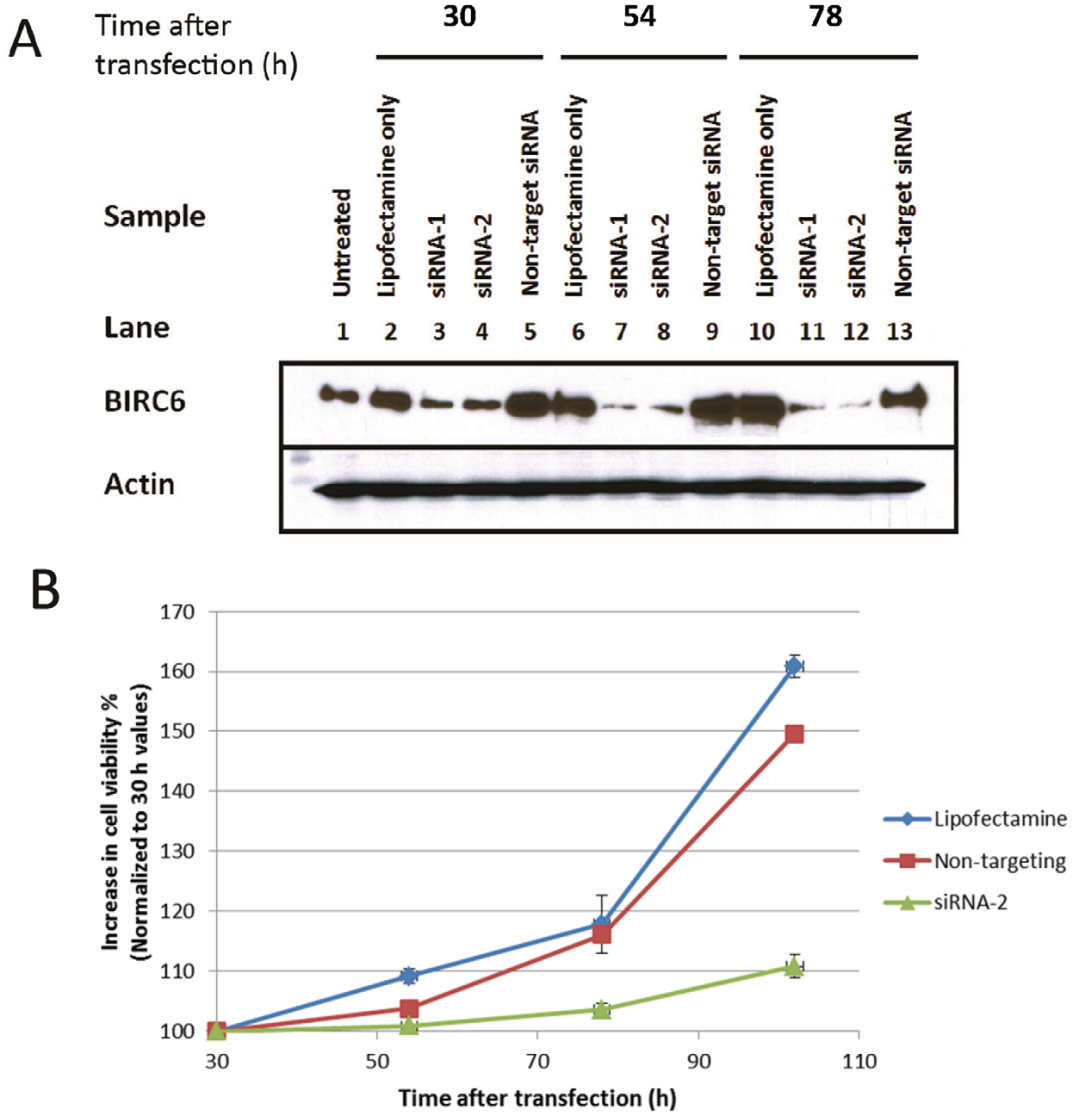
Reduction of BIRC6 expression decreases prostate cancer cell viability and proliferation. **A**, treatment of LNCaP cell cultures with siRNAs targeting BIRC6 leads to reduction of BIRC6 protein expression. BIRC6 protein expression in untreated LNCaP cells (Lane 1; at 78 h) and in LNCaP cells incubated with lipofectamine only (Lanes 2, 6, 10), siRNA-1 targeting BIRC6 (Lanes 3, 7, 11), siRNA-2 targeting BIRC6 (Lanes 4, 8, 12) or non-targeting siRNA (Lanes 5, 9, 13) for 30, 54 and 78 h after transfection. The results are representative of three independent experiments. **B**, treatment of LNCaP cell cultures with siRNA-2 targeting BIRC6 leads to reduced cell proliferation as shown by MTT assay. Cultures were treated for 30 h with lipofectamine only or with lipofectamine plus either non-targeting siRNA or siRNA-2 targeting BIRC6 and then incubated for 24, 48 and 72 h in fresh media. The relative cell numbers in the siRNA-2 cultures were considerably lower than those in the non-targeting siRNA treated cultures by 2.85%, 10.78% and 25.88% at 54, 78 and 102 h, respectively. Error bars depict standard deviations.

It has been reported that reduction of BIRC6 expression induces apoptosis (e.g., in breast cancer cells). We used the LNCaP cell line to study the effect of reducing BIRC6 expression on prostate cancer cell viability. Incubation of LNCaP cells transfected with *BIRC6*-targeting siRNA-1 and -2 (Lanes 3, 4, 7, 8, 11, 12) resulted in substantial loss of BIRC6 protein, compared to cells treated with lipofectamine only (Lanes 2, 6, 10), lipofectamine+non-targeting siRNA (Lanes 5, 9, 13) or no treatment (Lane 1) (Fig. 3A). The effect was apparent after 30 h of transfection and became more prominent at 54 and 78 h. It may be noted that cells transfected with lipofectamine only, or with lipofectamine+non-targeting siRNA, showed a small increase in BIRC6 expression, presumably due to the vehicle. All subsequent knockdown experiments were conducted using siRNA-2.

Following transfection, the siRNA-2 transfected cultures showed a marked reduction in cell viability relative to the non-targeting siRNA-treated cultures (Fig. 3B). Thus the cell viability of siRNA-2 cultures was considerably lower than that of the non-targeting siRNA-treated cultures by 2.85%, 10.78% and 25.88% at 54, 78 and 102 h, respectively. There was a tendency for the siRNA-2-treated cells to form syncytia-like structures in which clusters of cells were joined by long spindle-like projections. Results are representative of two independent experiments. The effect of BIRC6 silencing on cell cycle progression was also examined. The knockdown of BIRC6 in LNCaP cells did not result in significant change in cell cycling (Fig. S1).

The effect of BIRC6 reduction was also studied in PC-3 prostate cancer cells. Similar to LNCaP cells, siRNA-2 transfected PC-3 cells showed a significant decrease in cell viability compared to the non-targeting siRNA-treated cells 72 h after transfection (Fig. S2).

### Reduction of BIRC6 Expression Induces Apoptosis and Inhibits Autophagosome Formation

**Figure 4:**
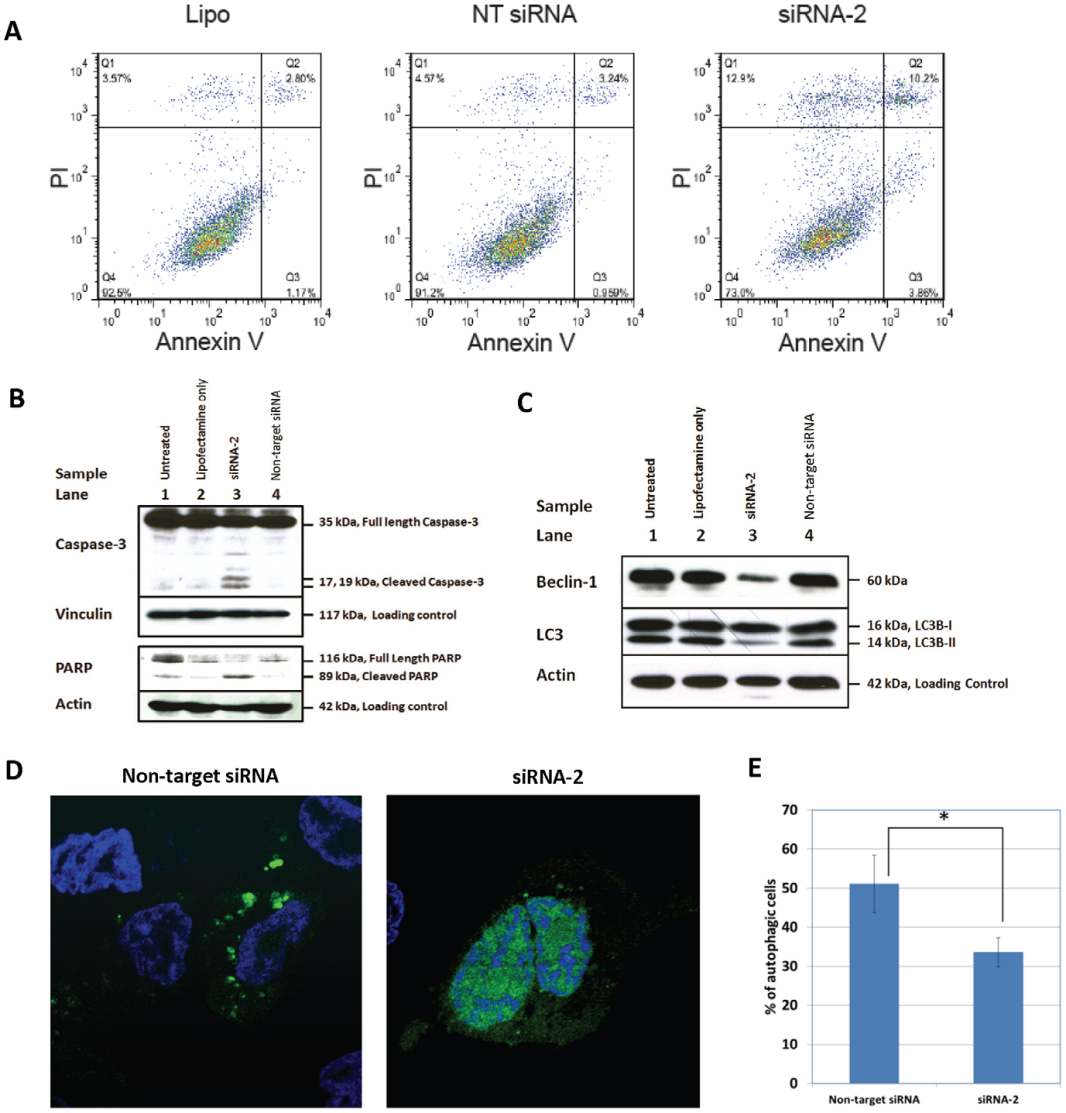
Decreased BIRC6 expression induces apoptosis and inhibits autophagosome formation. **A**, apoptosis of LNCap cells transfected with Lipofectamine (Lipo), non-targeting control siRNA (NT) or *BIRC6* siRNA2 and subsequently incubated for 48 h, as assessed by flow cytometric analysis of Annexin V/PI stained cells. On the basis of Q2+Q3 values (apoptotic cell populations), *BIRC6* siRNA markedly increased apoptosis of LNCaP cells compared with Lipo (p = 0.0104) or NT siRNA treated cells (p = 0.0123). (Data are representative of 3 independent experiments); **B**, treatment of LNCaP cells with siRNA-2 targeting BIRC6 leads to activation of caspase-3 (as shown by appearance of cleaved caspase-3) and degradation of its substrate, PARP (as shown by loss of full length PARP and appearance of a cleaved PARP product). Untreated LNCaP cells (Lane 1); LNCaP cells incubated with lipofectamine only (Lane 2), siRNA-2 targeting BIRC6 (Lane 3) or non-targeting siRNA (Lane 4) for 96 h following transfection. **C**, treatment of LNCaP cells with siRNA-2 targeting BIRC6 leads to decreases in Beclin-1 expression and reduction of LC3B-II. Untreated LNCaP cells (Lane 1); LNCaP cells incubated with lipofectamine only (Lane 2), siRNA-2 targeting BIRC6 (Lane 3) or non-targeting siRNA (Lane 4) for 113 h following transfection. **D**, autophagosome formation in BIRC6-silenced cells was significantly lower compared to cells treated with non-targeting control siRNA. Cells transfected with non-targeting siRNA control (left) showed more LC3-GFP puncta than cells transfected with siRNA-2 targeting BIRC6 (right), which showed diffused expression of LC3-GFP. **E**, Reduced BIRC6-expressing LNCaP cells shows significantly fewer autophagic cells (33.6%) compared to control cells (51%). Autophagic cells were quantified by counting cells with more than 15 LC3-GFP puncta under a confocal microscope.

BIRC6 reduction induces apoptosis in LNCaP cells as demonstrated by Annexin-V staining and immmunoblotting. As shown in Fig. 4A, there was a significant increase in Annexin-V positive cells in siRNA-2 transfected cells (12.19%±1.9%) compared to non-target siRNA (3.65%±0.60%, p = 0.0104) and Lipofectamine treated cells (3.55%±.0447%, p = 0.0123). Consistent with the Annexin-V assay results, BIRC6 knock-down cells showed marked changes in expression of apoptosis markers (Figure 4B). An increase in cleaved caspase-3, loss of full length PARP and an increase in a cleaved PARP were observed in comparison with LNCaP cells transfected with non-targeting siRNA (Lane 3, 4). Interestingly, we observed a full length PARP decrease following transfection of LNCaP cells with lipofectamine or non-targeting (control) siRNA (Lanes 2, 4), which might have been caused by non-specific degradation of full length PARP particularly in LNCaP cells. The loss of full length PARP in these controls was not associated with a corresponding increase in cleaved PARP and was not coupled to a significant increase in cleaved caspase-3, indicating that these controls did not result in induction of apoptosis.

Transfection of LNCaP cells with siRNA-2 (Lane 3) led to marked changes in autophagy marker expression (Fig. 4C). A decrease in LC3B-II protein (lipidated form of LC3-I) was observed with no effect on LC3B-I protein levels, as well as a decrease in Beclin-1 protein expression (compared with controls; Lanes 1, 2, 4). Moreover, autophagosome formation in BIRC6-silenced cells was significantly lower compared to cells treated with non-targeting control siRNA (*P* = 0.036) as revealed by fluorescent LC3 puncta accumulation in the cytoplasm (Fig. 4D, E). Taken together, the results show that reduction of BIRC6 protein expression in siRNA-2-treated LNCaP cells results in an increase in apoptosis and a decrease in autophagosome formation. Results are representative of three independent experiments.

### Doxorubicin-induced Apoptosis in LNCaP Cells is Associated with a Reduction in BIRC6 Protein Expression

**Figure 5:**
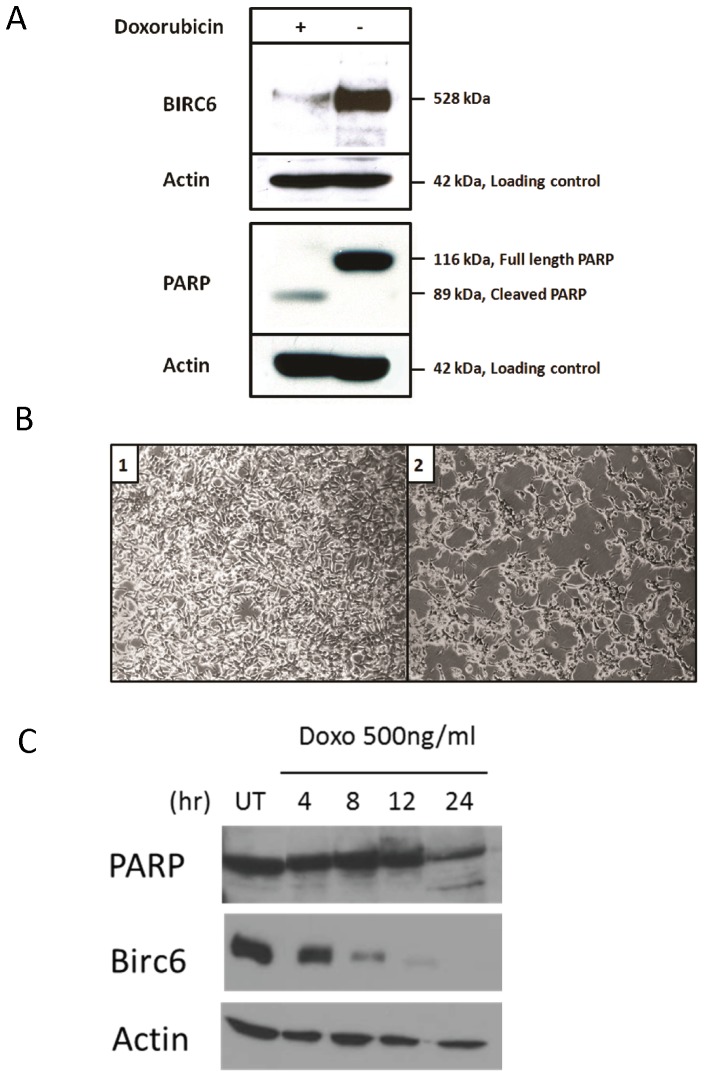
Doxorubicin-induced apoptosis in LNCaP cells is associated with a reduction in BIRC6 protein expression. **A**, incubation (24 h) of LNCaP cells with doxorubicin (1 µg/mL) leads to reduction of BIRC6 protein expression and apoptosis (loss of full length PARP and appearance of cleaved PARP) as indicated by Western blot analysis. **B**, LNCaP cells incubated for 24 h (1) without doxorubicin and (2) with doxorubicin (1 µg/mL) at 100× magnification. **C**, Expression of BIRC6 and PARP cleavage were studied in LNCaP cells incubated with doxorubicin (500 ng/mL) for 4, 8, 12, 24 h. BIRC6 expression was decreased upon treatment in a time-dependent manner and the reduction precedes apoptosis induction as indicated by PARP cleavage at 24 h.

Doxorubicin, a drug used for therapy of prostate cancer [32], has been reported to trigger apoptosis along with a marked accumulation of p53 [33]. To investigate the effect of doxorubicin-induced apoptosis on BIRC6 expression in prostate cancer cells, LNCaP cells were incubated for 24 h with or without doxorubicin (1 µg/mL). As shown by Western blot analysis, treatment with doxorubicin resulted in substantial reduction of BIRC6 expression (Fig. 5A). In addition there was a reduction in full length PARP protein expression and an increase in a cleaved PARP, indicative of apoptosis. Furthermore, there was a reduction in cell density with evidence of cell deterioration (Fig. 5B). Treatment of LNCaP cells with doxorubicin showed a dose- and time-dependent reduction of BIRC6 expression (Fig. S3). To understand whether BIRC6 reduction was a cause or a result of doxorubicin induced-apoptosis, temporal expression of BIRC6 and PARP after doxorubicin treatment was studied. BIRC6 protein expression decreased continuously beginning from 4 hours to 24 hours after treatment with doxorubicin at 500 ng/mL. The reduction of BIRC6 was marked at 8 hours, yet PARP cleavage was not observed until 24 hours after treatment, suggesting that BIRC6 reduction is an early response to doxorubicin but not the result of doxorubicin-induced apoptosis (Fig. 5C).

## Discussion

BIRC6 has been reported to play a significant role in apoptosis resistance of a variety of cancers [21], [23]–[27]. In the present study we investigated whether it also plays a role in apoptosis resistance of prostate cancer, as this process may underlie the development of castration resistance. In contrast to earlier reports [22], our study established that the BIRC6 protein is markedly expressed by a variety of conventional malignant prostate cell lines as distinct from benign prostate cell lines (Fig. 1), indicating that BIRC6 could have a significant role in prostate cancer.

BIRC6 was found to be functionally critical for the survival of prostate cancer cells. Specific reduction of BIRC6 expression by siRNAs led to a marked inhibition of prostate cancer cell viability (Fig. 3, S2), which notably was coupled to a marked increase in Annexin-V positive cells and the expression of apoptosis markers (Fig. 4A, B). The reduction in population growth induced by non-targeting siRNA is likely due to non-specific toxicity as reported by others [34]; importantly, it was not associated with an increase in apoptosis marker expression. The results are consistent with reports of a critical role for BIRC6 in the survival of a variety of cancer cells [21], [23]–[26]. Cell cycle analysis showed that BIRC6 reduction did not result in significant change in cell cycle distribution (Fig. S1), suggesting that the reduction in cell viability was attributable to apoptosis.

In this study, a decrease in cell viability induced by BIRC6 reduction did not confine to cells expressing wild-type *p53*, contrary to previous reports suggesting that apoptosis resulting from BIRC6 knockdown in H460 cells and breast cancer cells requires functional p53 [23], [25]. We showed that both wild type p53 (LNCaP) and p53 null (PC-3) cells were sensitive to BIRC6 siRNA induced growth inhibition. This variation reflects that apoptosis induction by loss of BIRC6 may be facilitated by different mechanisms in different models. Further investigation is necessary to understand the underlying mechanism leading to apoptosis after BIRC6 reduction in p53 null cells and that will provide further insight in the possibility of targeting BIRC6 in cancer cells lacking functional p53.

Elevated levels of BIRC6 have been linked to apoptosis resistance, for instance in the SNB-78 glioma cell line [22] and over-expression of BIRC6 in human fibrosarcoma cells supports resistance to anti-cancer drugs and death receptor ligation [18]. Furthermore, down-regulation of BIRC6 expression in SNB-78 cells was shown to sensitize the cells to apoptosis induced by cisplatin and camptothecin [22]. It is therefore conceivable that the elevated expression of BIRC6 observed in castration-resistant prostate cancers (Fig. 2F) may be responsible for the treatment resistance of refractory disease.

The specific reduction of BIRC6 expression in LNCaP cells leading to a decrease in the expression of LC3B-II and Beclin-1 (Fig. 4C) and decline in autophagosome accumulation (Fig. 4D, E), suggest that there is a novel role for BIRC6 in the regulation of autophagy.

The reduced expression of LC3B-II indicates that loss of BIRC6 expression results in a lower number of autophagosomes. However, based on LC3B-II levels alone, it is not possible to determine whether the reduced number of autophagosomes is due to a decrease in autophagosome formation or to an increase in autophagosome degradation. To provide further insight into regulation of autophagy by BIRC6, BIRC6-depleted LNCaP cells were also examined for changes in the levels of Beclin-1 (Fig. 4C). In mammalian cells enhanced expression of Beclin-1 has been shown to increase their autophagic response [35] and the suppression of Beclin-1 has been shown to impair autophagy and sensitize cells to starvation-induced apoptosis [36]. The reduced expression of Beclin-1 in the BIRC6-depleted LNCaP cells suggests that the lower numbers of autophagosomes in these cells is likely due to inhibition of autophagy initiation and autophagosome formation.

In addition, in the LC3-GFP puncta formation assay where LNCaP cells were treated with chloroquine, a potent inhibitor of autophagosome degradation, BIRC6-depleted cells also showed less punctate structures than non-targeting controls. This evidence highlights the relationship between BIRC6 expression and autophagy initiation in particular. Taken together, these data demonstrate that loss of BIRC6 expression in LNCaP prostate cancer cells leads to inhibition of autophagy and that BIRC6 may be a positive regulator of autophagy.

With increasing evidence that autophagy may serve as a survival mechanism of cells in response to stress, including anti-cancer therapeutics [7], BIRC6 may be a suitable target for inhibition of autophagy-mediated cell survival and for treatment resistance in prostate cancer cells. Targeting autophagy has already been shown to sensitize a variety of cancers to treatment, including prostate cancer [37], [38]. Treatment of prostate cancer cells deficient in argininosuccinate synthetase with siRNAs targeting *Beclin-1* or chloroquine (an autophagy inhibitor), has been reported to inhibit autophagy and increase the sensitivity of such cells to treatment with the anti-cancer agent ADI-PEG20, a pegylated arginine deiminase [37]. In view of the above, it is proposed that targeting BIRC6 in prostate cancer can be used to inhibit autophagy, and thus, autophagy-mediated treatment resistance. This strategy represents a novel approach to sensitizing prostate cancer cells to therapy. However, further work is needed to determine the effectiveness of targeting BIRC6 as a strategy to control autophagy-mediated treatment resistance.

The finding that treatment of LNCaP cells with doxorubicin results in a dramatic loss of BIRC6 expression (Figs. 5A, C, S3), is consistent with a previous report demonstrating that apoptosis induced by topoisomerase inhibitors etoposide and camptothecin was associated with degradation of BIRC6 protein [25]. The authors concluded that degradation of BIRC6 appears to be a general event during initiation of apoptosis [25]. In the present study we further demonstrated that the doxorubicin-induced BIRC6 decline precedes PARP cleavage, suggesting that BIRC6 may play a causative role in apoptosis induction upon doxorubicin treatment (Fig. 5C). In addition, our finding that specific siRNA-induced reduction of BIRC6 protein expression in LNCaP cells leads to apoptosis, as indicated by marker expression (Fig. 4B), raises the possibility that the apoptotic effect of doxorubicin and perhaps of the topoisomerase inhibitors, is based, at least in part, on a reduction of BIRC6 protein expression. This suggests a novel mechanism by which doxorubicin may induce apoptosis by triggering loss of BIRC6.

The increase in BIRC6 expression in Gleason 6–8 clinical prostate cancers (Fig. 2E), including castration-resistant cancers (Fig. 2F), suggests an important role for this protein in the development and progression of the disease. In view of the pro-survival function of BIRC6 in prostate cancer cells (Figs 3B, 4A, B) and in other systems [21], [23]–[26], elevations in the expression of BIRC6 are expected to provide a cytoprotective advantage to prostate cancer cells and promote prostate cancer development and progression. The anti-apoptotic role of BIRC6 could likely be involved in the development of castration-resistant prostate cancer and underlie therapy resistance in this advanced form of the disease. While the large majority of prostate cancer tissues exhibited BIRC6 protein elevations, not all stages of the disease expressed elevated levels of the protein. The expression of BIRC6 over the course of prostate cancer progression reached peak levels in Gleason score 7 cancers but had levels in Gleason score 9–10 prostate cancers that were similar to those of benign tissues (Fig. 2E). Our finding is consistent with an earlier study focusing on IAP expressions in various stages of prostate cancer tissues, which demonstrated that increased expression of IAP (cIAP-1, cIAP-2, XIAP and survivin was an early event in prostate cancer and did not correlate with Gleason grade [39] Our data and previous studies suggested that BIRC6, like other IAP family members, is functionally more critical at an early stage than at a late stage of prostate cancer.

On the other hand, the levels of BIRC6 protein were elevated in castration-resistant Gleason 8–10 prostate cancers compared to non-castrated counterparts (Fig. 2F). While BIRC6 expression may not be required in advanced stage prostate cancer, the resurge of BIRC6 in CRPC may suggest that cellular stress, e.g. castration, may trigger the overexpression of cytoprotective BIRC6. Further investigation is necessary to address the cause for the change. Nevertheless, the elevation of BIRC6 in castration-resistant cancers suggests that the protein may provide a potential therapeutic target for the disease.

Targeting BIRC6 as an inhibitor of apoptosis and potential enhancer of autophagy may be useful for sensitizing prostate cancer cells to anti-cancer therapies. It may be noted that drugs targeting other IAP family members, e.g., XIAP and survivin, have shown promise for use as sensitizers in prostate cancer therapy. Antisense inhibitors of XIAP led to sensitization of castration-resistant prostate cancer cells to cisplatin and TNF-related apoptosis-inducing ligand (TRAIL) [40]; in PC3 prostate cancer xenografts, they caused sustained tumor regression in combination with docetaxel [41].

In conclusion, the present study indicates for the first time that the *BIRC6* gene and its product are potentially valuable targets for treatment of prostate cancers showing elevated BIRC6 expression. Notably, BIRC6 may provide a novel therapeutic target for the treatment of castration-resistant disease.

## Supporting Information

Figure S1 **Cell cycle analysis of (A) mock, (B) non-targeting control siRNA and (C) BIRC6-targeted siRNA transfected LNCaP cells.** BIRC6 knockdown (siRNA-2) did not resulted in significant change in cell cycle at 48 h after transfection. (D) Percentage of cell population at sub-G1, G1, S and G2/M phase cells. Results were shown as mean of triplicate experiments.
(TIFF)

Figure S2 **A, Western blot shows apparent decrease of BIRC6 expression at 72 h after siRNA transfection in PC-3 cells. Cells were transfected with 100 nM NT siRNA or siRNA-2 by Oligofectamine; B, Knock-down of BIRC6 in PC-3 cells resulted in significant reduction of cell viability at 72 h after transfection by MTT assay (p<0.01).**
(TIFF)

Figure S3 **Doxorubicin treatment down-regulates BIRC6 protein expression in a dose- and time-dependent manner.**
(TIFF)
