# Supplementary figures and images for: BIRC6 Protein, an Inhibitor of Apoptosis: Role in Survival of Human Prostate Cancer Cells
Source: PLoS One. 2013 Feb 8;8(2):e55837. doi: 10.1371/journal.pone.0055837 (PMC3568134; doi:10.1371/journal.pone.0055837)

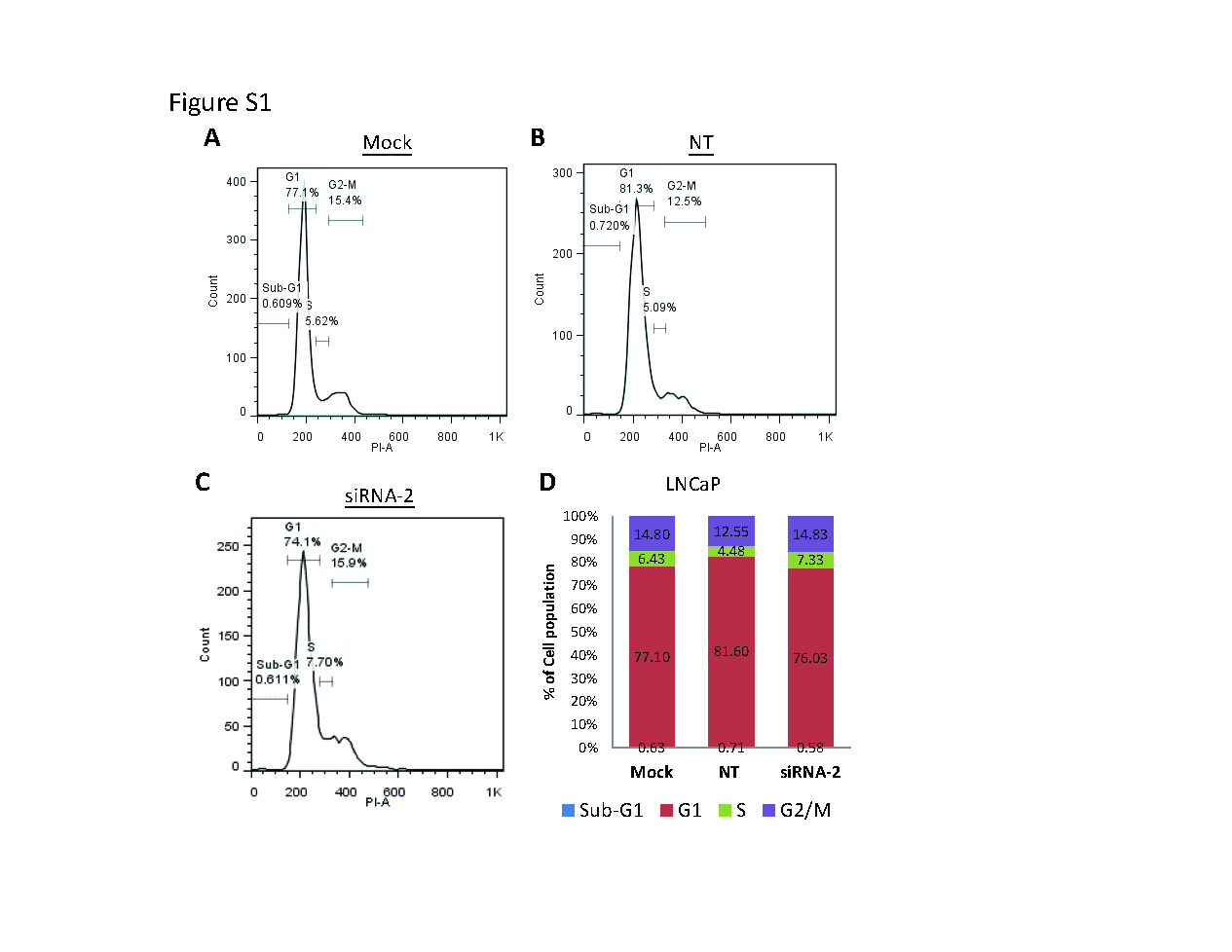

Supplement: Figure S1 — Cell cycle analysis of (A) mock, (B) non-targeting control siRNA and (C) BIRC6-targeted siRNA transfected LNCaP cells. BIRC6 knockdown (siRNA-2) did not resulted in significant change in cell cycle at 48 h after transfection. (D) Percentage of cell population at sub-G1, G1, S and G2/M phase cells. Results were shown as mean of triplicate experiments. (TIFF) [file pone.0055837.s001.tiff]

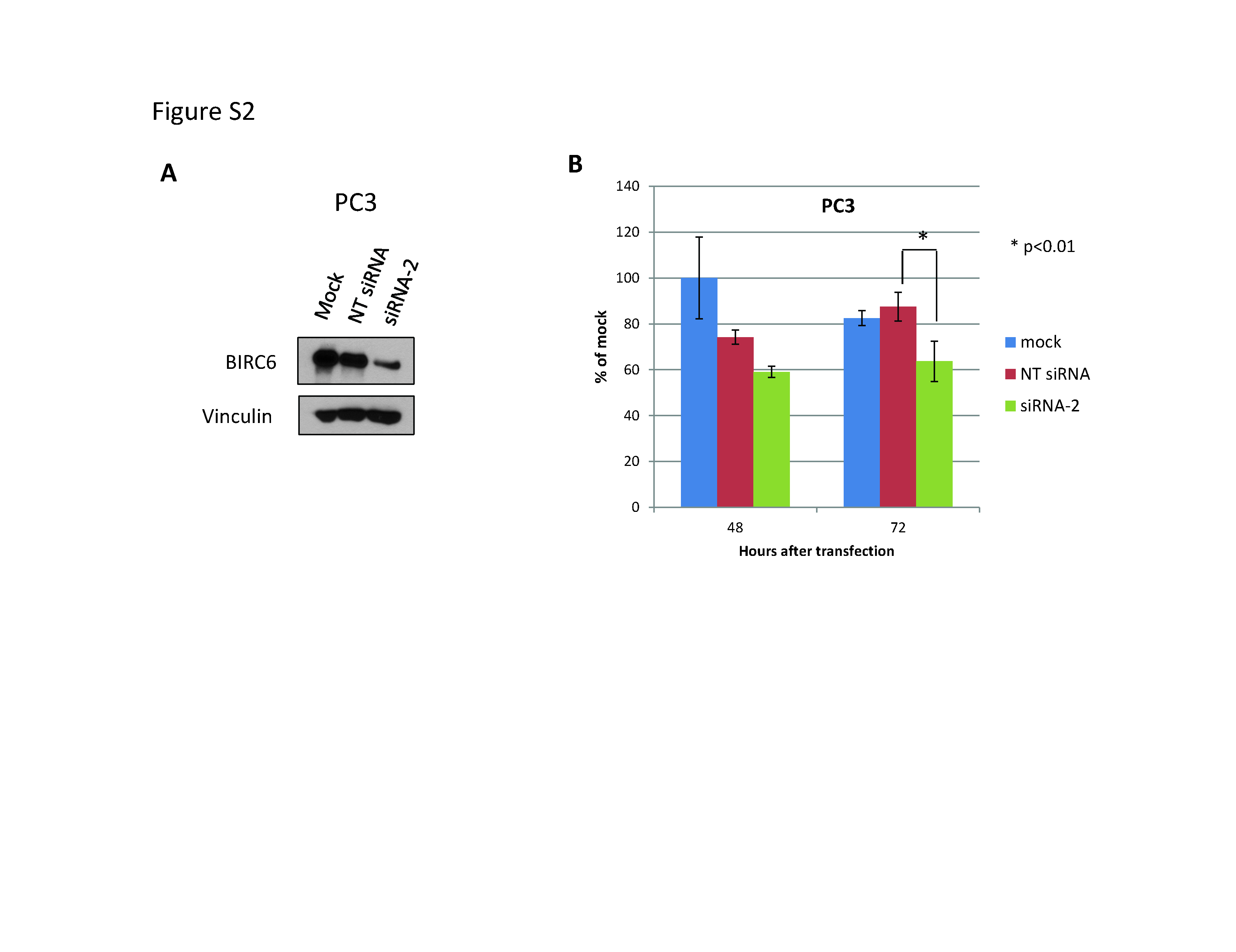

Supplement: Figure S2 — A, Western blot shows apparent decrease of BIRC6 expression at 72 h after siRNA transfection in PC-3 cells. Cells were transfected with 100 nM NT siRNA or siRNA-2 by Oligofectamine; B, Knock-down of BIRC6 in PC-3 cells resulted in significant reduction of cell viability at 72 h after transfection by MTT assay (p<0.01). (TIFF) [file pone.0055837.s002.tiff]

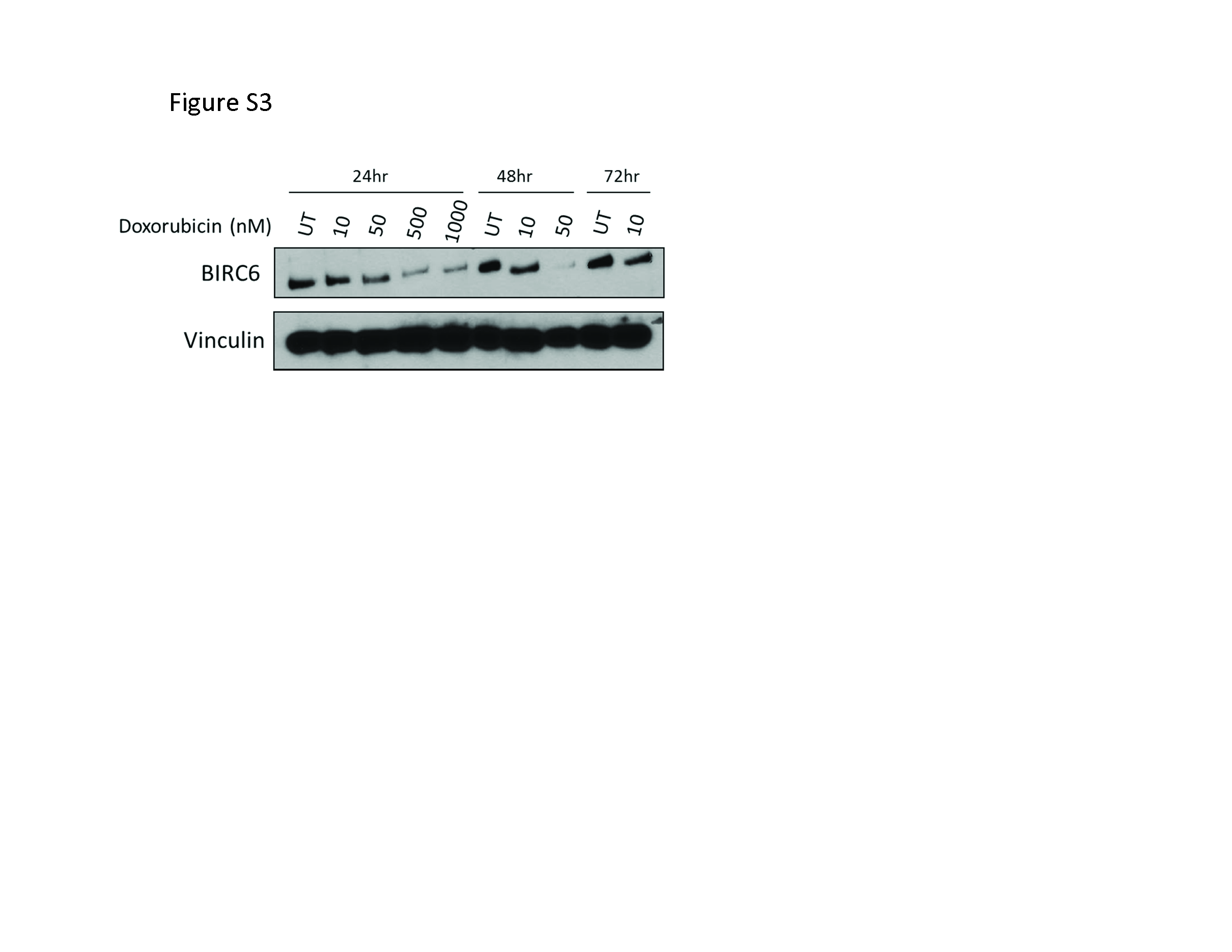

Supplement: Figure S3 — Doxorubicin treatment down-regulates BIRC6 protein expression in a dose- and time-dependent manner. (TIFF) [file pone.0055837.s003.tiff]
